# Real-World Implementation and Evaluation of a Digitally Enabled Care Pathway to Improve Depression Management in Primary Care: Controlled Pre-Post Analysis of the Pathway Platform

**DOI:** 10.2196/93024

**Published:** 2026-08-06

**Authors:** Rasha Khatib, Christopher Blair, Maggie McCue, Ben Fehnert, James King, Lara Zaki, Lambros Chrones, Anit Roy, Michael L Martin, Ramona Donovan, Marybeth Ingle, Iridian Guzman, David E Kemp

**Affiliations:** 1 Advocate Aurora Research Institute Milwaukee, WI United States; 2 Takeda Pharmaceuticals U.S.A., Inc. Lexington, MA United States; 3 Cognition Kit Cambridge United Kingdom; 4 Ctrl Group/Fora Health London United Kingdom; 5 Advocate Health Care Downers Grove, IL United States

**Keywords:** major depressive disorder, MDD, digital health, patient participation, patient health questionnaire, measurement-based care, shared decision-making

## Abstract

**Background:**

Measurement-based care (MBC) and shared decision-making can improve clinical outcomes for patients with major depressive disorder (MDD) in primary care. However, primary care providers often have limited time to administer patient-reported outcome measures recommended for MBC. Digital health tools like the Pathway Platform can help facilitate MBC and patient-provider engagement, aiding enhanced shared decision-making.

**Objective:**

This study aimed to assess whether implementation of the Pathway Platform improves adherence to MBC practices in primary care clinics compared with standard care. Secondary objectives included whether implementation of Pathway Platform improves additional clinical process measures and MDD remission and response.

**Methods:**

This real-world, longitudinal implementation study screened participants aged ≥18 years with MDD who started an antidepressant or switched within 3 months in primary care clinics in Illinois. Data were collected 6 months retrospectively (from the preimplementation period) using electronic health records and manual chart reviews (control cohort), and 6 months prospectively (postimplementation, conducted 2021 to 2023) using electronic health records, manual chart reviews, and the Pathway Platform mobile app (Pathway cohort). The primary outcome was the proportion of participants with ≥2 completed 9-Item Patient Health Questionnaires (PHQ-9) at 6 months. Secondary outcomes included health care resource usage, patient-provider engagement (13-Item Patient Activation Measure [PAM-13]), and overall functioning (Work and Social Adjustment Scale [WSAS]). Exploratory outcomes included cognitive functioning (5-Item Perceived Deficits Questionnaire–Depression [PDQ-D-5]) and MDD symptom improvement.

**Results:**

The control cohort had 90 participants (58% female), and the Pathway cohort had 89 (80% female). A significantly higher proportion of Pathway cohort participants completed ≥2 PHQ-9s than control cohort participants (19%, n=17 vs 12%, n=11; *P*=.01). All Pathway cohort participants completed ≥1 PHQ-9 assessment, and 45% (n=40) of participants opened the app at least once per week. Pathway participants were significantly less likely than controls to have behavioral health referrals (9%, n=8 vs 23%, n=21; *P*=.01). Patient-provider engagement assessed using PAM-13 showed significant improvement from baseline to 6 months among Pathway participants (n=54; median change from baseline 4, Q1 0 to Q3 9.2], 95% CI 0-4.9; *P<*.001). Median change from baseline to last available scores for WSAS (–4.0, Q1 –9 to Q3 1; *P*<.001) and PDQ-D-5 (–2, Q1 –6 to Q3 0; *P*<.001) showed improvement among patients in the Pathway cohort. Median PHQ-9 total score at 6 months was significantly lower in the Pathway cohort than the control cohort (6.5, Q1 3.0 to Q3 12.5 vs median 17.0, Q1 14.5 to Q3 20.0; *P*=.02), indicating improvement in MDD symptoms.

**Conclusions:**

The Pathway Platform significantly improved PHQ-9 completion rates, patient-provider engagement (improved PAM-13 scores), and clinical outcomes for MDD, with fewer behavioral health referrals. These findings suggest that use of the Pathway Platform supports improved MBC, offering a promising tool to improve MDD outcomes in primary care settings.

**Trial Registration:**

ClinicalTrials.gov NCT04891224; https://clinicaltrials.gov/ct2/show/NCT04891224

**International Registered Report Identifier (IRRID):**

DERR1-10.2196/43788

## Introduction

With an overall prevalence of 8.3% among adults in 2021, major depressive disorder (MDD) is one of the most common mental disorders in the United States, causing a high socioeconomic burden and disrupting daily activities as well as quality of life [1-3]. Although many antidepressant treatments are available, people with MDD may experience remission or relapse after acute therapy, requiring long-term management [4,5]. The American Psychiatric Association (APA) guidelines for the treatment of depression recommend periodic monitoring for benefit as measurement-based care (MBC) every 2 weeks during the acute management phase or 6 weeks during long-term care [4,6]. Patient-reported outcome (PRO) tools are validated methods to evaluate patients’ responses to treatment and can support shared decision-making (SDM) between clinicians and their patients through individualized therapy plans [4,6]. Therefore, incorporating MBC and SDM in patient care can improve treatment outcomes [4,6].

In the United States and around the world, most patients with depression are treated in the primary care setting and are not referred to specialists [4,7]. Yet, primary care providers (PCPs) often have limited time to administer the PRO tools recommended for MBC [8,9].

Digital technology platforms and mobile apps can facilitate MBC and SDM through scheduled collection of PROs and can relay results to the clinical care team through the patient’s electronic health records (EHRs) [6,10,11]. Although many apps for the management of MDD have been tested in the research setting, very few have been integrated into the clinical workflow [6,12].

The digitally enabled Pathway Platform is a health-technology experience for patients and health care providers (HCPs) [6]. For patients, the Pathway Platform is available as a smartphone app with a conversational texting interface to record daily symptom changes and responses to antidepressant treatment [6]. Integrated into the user’s or patient’s EHR, the platform captures routinely used PROs, such as the 9-Item Patient Health Questionnaire (PHQ-9), to monitor changes in symptoms and severity of MDD [6].

Previously, a collaborative, randomized controlled pilot study demonstrated that patients with MDD engaged with the Pathway Platform, which facilitated the systematic use of MBC for MDD management in primary care, establishing the feasibility and effectiveness of the Pathway Platform in this setting [13]. In another qualitative study, patient interviews and thematic analysis identified needs among the systems, patients, and care team to guide development of the current version of the Pathway Platform (developed by Takeda Pharmaceuticals U.S.A., Inc., Lundbeck LLC, and Advocate Aurora Health [AAH]; powered by Ctrl Group/Fora Health), contributing to a cocreated Pathway Platform model [10]. The current iteration of the Pathway Platform includes real-time data sharing and improved MDD-related education for patients and care teams [10]. Here, we report on the implementation of the Pathway Platform in a primary care clinical setting in the United States. We evaluated whether use of the Pathway Platform by patients with MDD and their HCPs improved depression management, patient-provider engagement, and MDD-related clinical outcomes compared with a preimplementation control cohort.

## Methods

### Study Design

This prospective implementation study (ClinicalTrials.gov NCT04891224) used a pre- and poststudy design.

The study was divided into the preimplementation period, implementation period, and postimplementation period. The preimplementation period was prespecified to occur at least 6 months before implementing the Pathway Platform. A control cohort was selected consisting of participants who met the eligibility criteria and had attended a participating primary care clinic during the preimplementation period. Data for the control cohort were collected retrospectively using EHRs and manual chart reviews, allowing for 6 months of follow-up. During the implementation period, participants were trained and onboarded to the Pathway Platform, including the app-based educational program. Additionally, PCPs received education on MBC, SDM, and evidence-based clinical practices for depression care through the web-based educational training program. In the postimplementation period data from the Pathway cohort were prospectively collected over 6 months using EHRs, manual chart reviews, and the Pathway Platform [6].

### Ethical Considerations

The Study was Approved by the AAH Institutional Review Board (IRB Number: IRB00106913).

This real-world longitudinal study was conducted through the AAH system, in which PCPs from 8 AAH primary care sites in Illinois were invited to participate in the study. These primary care sites were selected throughout the AAH system to best represent the real-world patient population with MDD.

Following referral from a participating physician, a research team member contacted the patient by phone to explain the study in detail. The research team member emailed the informed consent form to the patient using the REDCap (Vanderbilt University) secure web platform and then reviewed it with the patient during the phone call. Patients who agreed to participate submitted signed consent forms through REDCap, where documents were securely stored. Study participants received a US $75 gift card at the end of the 6-month study period in compensation for their time and effort. To ensure confidentiality, study data were extracted from electronic medical records (EMRs) and chart reviews, entered into a password-protected Excel file, and accessed only by authorized staff. Each participant was assigned a unique identification number. Data were securely stored (SOC2-compliant) and transmitted among the Pathway Platform, servers, and EMR systems; connectivity between the Pathway Platform and EMRs met SMART (Substitutable Medical Applications, Reusable Technologies) specifications for Fast Healthcare Interoperability Resources standards. Identifiable links were destroyed at study close. The study data and results are the property of Takeda Pharmaceuticals US, Inc. The software product used to support the study is the exclusive property of Fora Health Ltd. This study was reported in accordance with the iCHECK-DH (Guidelines and Checklist for the Reporting on Digital Health Implementations) and STROBE (Strengthening the Reporting of Observational Studies in Epidemiology) reporting guidelines [14,15].

### Study Population

Eligibility criteria for both cohorts included adult patients (aged ≥18 years) with a diagnosis of MDD who had visited 1 of the 8 participating AAH primary care sites in Illinois within the designated study period, had recently started an antidepressant or changed antidepressant therapy within 3 months, and had an inadequate response based on a 2-Item Patient Health Questionnaire (PHQ-2) score of ≥3 or a PHQ-9 score of ≥5, while receiving care from a participating PCP. Patients diagnosed as having bipolar disorder, schizophrenia, or schizoaffective disorder were excluded from the study. A detailed study description was previously published [6].

### Study Intervention

The Pathway cohort participants received the version of the Pathway Platform updated after the pilot study, which consisted of 3 components: the mobile Pathway app, EHR integration, and educational scaffolding. The app prompts completion of 4 PRO scales every 2 weeks: PHQ-9 to assess depressive symptoms [16], 5-Item Perceived Deficits Questionnaire–Depression (PDQ-D-5) to assess cognitive symptoms [17], 5-Item World Health Organization Well-Being Index (WHO-5) for overall well-being [18], and a cognitive assessment using the Cognition Kit Digit Symbol Substitution Test (DSST; Table S1 in Multimedia Appendix 1) [19]. Other scales integrated for use at baseline and end of study were the 13-Item Patient Activation Measure (PAM-13) [20] to assess patient-provider engagement, and the Work and Social Adjustment Scale (WSAS) [21] for assessment of daily functioning. A daily check-in for any medication-associated adverse events (AEs) included prompts for decline in sleep quality and sexual well-being. If participants reported one or both of these AEs, the app initiated the Patient-Reported Outcomes Measurement Information System (PROMIS) Sleep Disturbance Item 6a or the Arizona Sexual Experience Scale (ASEX). Goal setting and tracking was an optional feature for participants.

### Study Objectives and Outcomes

The primary objective of the study was to evaluate whether implementing the Pathway Platform in primary care improves adherence to MBC, by examining PHQ-9 usage over 6 months in the control and Pathway cohorts. The PHQ-9 is a widely validated quantitative tool for assessing depressive symptom severity and treatment response and is integrated into the Pathway app [16]. Periodic assessment of MDD severity through PHQ-9 is in alignment with clinical guidelines that describe MBC, including Healthcare Effectiveness Data and Information Set (HEDIS) and APA guidelines [4,6,22,23]. Secondary objectives of the study included determining whether implementing the Pathway Platform improves additional clinical process measures and MDD response and remission outcomes.

The primary outcome was the proportion of participants with ≥2 available PHQ-9 scores in the EHRs at 6 months. In addition, we compared PHQ-2 usage rates at 6 months because an analysis of EHR data showed that many patients had scores for this assessment, because clinicians completed the PHQ-2 scale as part of the clinical workflow. Secondary outcomes included the proportion of participants with MDD medication changes during the 6-month observation period, an indication of MBC, and proportions of participants who received a referral to behavioral health, were hospitalized for mental health, or visited an emergency department (ED) for mental health. Additional secondary outcomes included MDD remission and response rates and the difference between baseline and 6 months for the PAM-13 and WSAS scores. MDD remission was defined as a PHQ-9 score of ≤5, and MDD response was defined as a ≥50% reduction in PHQ-9 score (most recent score). Exploratory outcomes included improvement in MDD severity from baseline to 6 months as measured by PHQ-9 scores; change in PHQ-9, WHO-5, and PDQ-D-5 scores between the first and last score; app engagement; and medication-associated AEs reported by patients through the app.

### Statistical Analysis

Based on the primary outcome of PHQ-9 usage, it was estimated that 100 participants per group would be needed to achieve 75% usage prior to Pathway implementation and 90% usage after implementation, assuming 80% power and a 2-sided alpha of .05 using Pearson’s chi-square test for 2 independent proportions. For comparisons of the primary and secondary outcomes between the Pathway and control cohorts, ordinal variables were compared using a Wilcoxon test, and categorical outcomes, presented as proportions of participants, were compared using Pearson’s chi-square test. Exploratory analysis included change in outcomes over time, and categorical outcomes, presented as proportions of participants, were compared using McNemar test. For all analyses, continuous outcomes, checked for normality and presented as the median with Q1 and Q3, were compared using a Wilcoxon test. A mixed-effects model for repeated measures adjusted for age, sex, and baseline PHQ-9 was used to compare the difference in mean change in PHQ-9 scores from baseline to 3 months and to 6 months, comparing the preimplementation control cohort and postimplementation Pathway cohort. All statistical tests were 2-tailed, using a significance threshold of .05. All statistical tests were performed using SAS statistical software suite 9.4 (SAS Institute Inc).

## Results

### Participant Disposition and Baseline Characteristics

#### Patient Populations

In the preimplementation period, 1271 patients who visited participating clinics in Illinois had an MDD diagnosis. Of these, we identified 90 patients who were eligible and were included in the control cohort. In the postimplementation period, conducted between March 2021 and July 2023, we prospectively identified 192 patients (of 2772 screened) who were eligible and then invited to participate as part of the Pathway cohort. From that group, 89 gave consent to participate and activated the Pathway Platform app (Figure S1 in Multimedia Appendix 1).

#### Baseline Demographics and Disease Characteristics

The Pathway cohort had a younger median age (*P*=.03) and a higher proportion of female participants compared with the control cohort (*P*<.001; Table 1). The percentage of non-Hispanic White participants was significantly lower in the Pathway cohort compared with the control cohort (*P*=.01); correspondingly, the percentage of non-Hispanic Black participants was significantly higher in the Pathway cohort compared with the control cohort (*P*=.01). Baseline PHQ-9 scores were generally balanced between the Pathway and control cohorts, indicating that the proportions of participants with mild to moderate MDD (54/90, 60% in control cohort and 47/89, 53% in Pathway cohort) and moderately severe to severe MDD (36/90, 40% in control cohort and 42/89, 47% in Pathway cohort) were similar.

**Table 1:** Patient demographic and clinical characteristics at baseline^a^ in each cohort.

Characteristics			Control cohort (preimplementation period; n=90)		Pathway cohort (postimplementation period; n=89)		*P* values	
Age, median (Q1, Q3), (years)			39 (28, 57)		35 (28, 46)		.03	
Female sex, n (%)			52 (58)		71 (80)		<.001	
**Race or ethnicity,** **n (%)**								.01
	Non-Hispanic White	50 (56)		41 (46)				
	Hispanic White	16 (18)		16 (18)				
	Non-Hispanic Black	14 (15)		29 (33)				
	Other	10 (11)		3 (3)				
**Type of insurance, n (%)**								.20
	Medicare	14 (15)		6 (7)				
	Commercial	59 (66)		68 (76)				
	Medicaid	17 (19)		15 (17)				
Baseline PHQ-9^b^ score, median (Q1, Q3)			14 (11, 17)		15 (12, 18)		.14	
Mild to moderate (score <15), n (%)			54 (60)		47 (53)			
Moderately severe to severe (score ≥15), n (%)			36 (40)		42 (47)			
**Medications, n (%)**								
	SSRIs^c^	69 (77)		67 (75)		.83		
	SNRIs^d^	7 (8)		3 (3)		.33		
	Alpha-2 receptor antagonists (tetracyclics); mirtazapine	2 (2)		1 (1)		≥.99		
	Tricyclic agents	0		1 (1)		.50		
	Serotonin modulators	1 (1)		0		≥.99		
	Bupropion	11 (12)		17 (19)		<.001		

^a^Baseline data sourced from the electronic health record.

^b^PHQ-9: 9-Item Patient Health Questionnaire.

^c^SSRI: selective serotonin reuptake inhibitor.

^d^SNRI: serotonin–norepinephrine reuptake inhibitor.

### Primary Outcome: Use of PHQ Assessments

**Figure 1:**
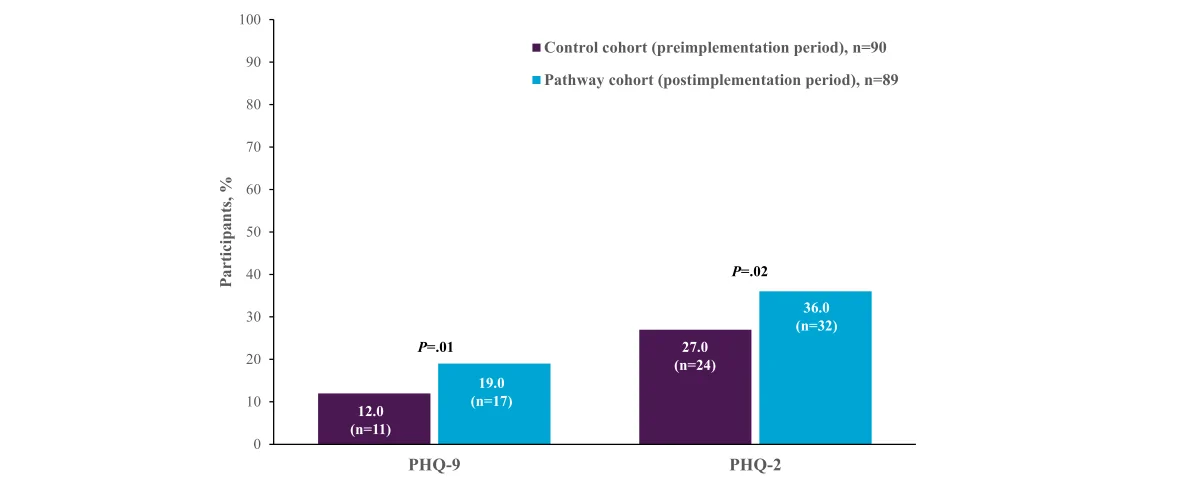
Participants with ≥2 Patient Health Questionnaire-9 or Patient Health Questionnaire-2 assessments preimplementation (control cohort) and postimplementation (Pathway cohort) of the Pathway Platform. Group comparison: Pearson's chi-square test. Sourced from electronic health record data. PHQ-2: 2-Item Patient Health Questionnaire; PHQ-9: 9-Item Patient Health Questionnaire.

A significantly higher proportion of participants in the Pathway cohort completed ≥2 PHQ-9 assessments compared with the control cohort (19%, 17/89 vs 12%, 11/90; *P*=.01; Figure 1). Similarly, significantly more Pathway participants than controls had PHQ-2 scores during the 6-month study (*P*=.02). The proportion of participants with mild to moderate MDD who had ≥2 PHQ-2 or PHQ-9 assessments was significantly higher in the Pathway cohort than in the control cohort (57%, 27/47 vs 35%, 19/54; *P*=.03). There was no difference in the use of the PHQ scales among participants with moderately severe to severe MDD between the 2 cohorts (Table S2 in Multimedia Appendix 1).

### Secondary and Exploratory Outcomes

#### Management of MDD

**Figure 2:**
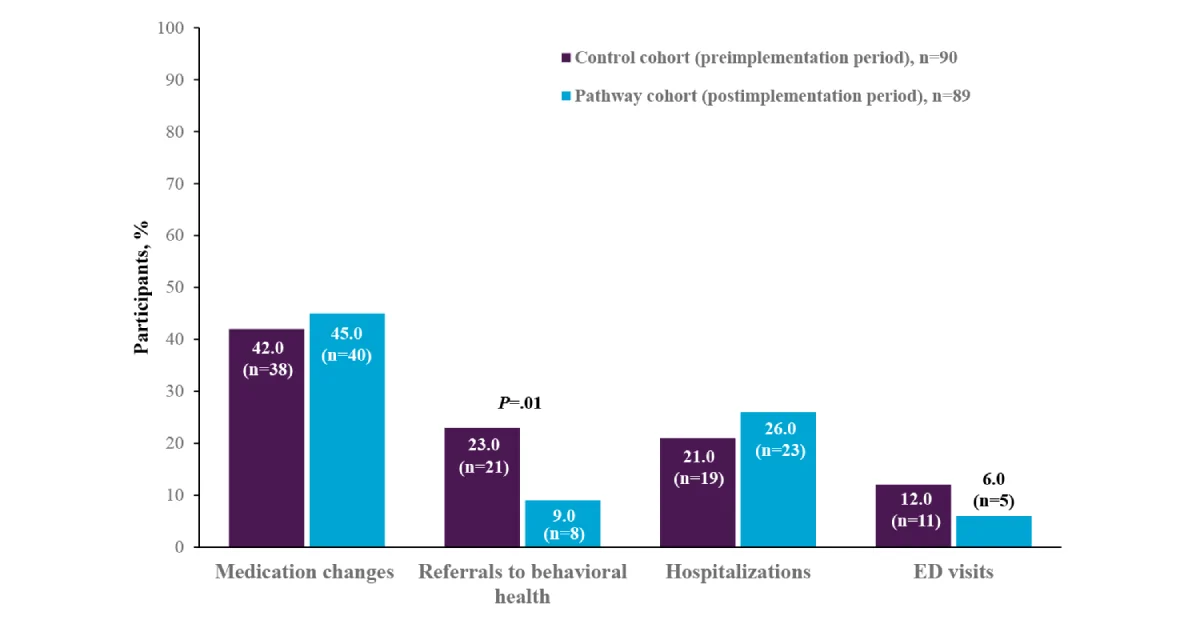
Management of major depressive disorder over the 6-month preimplementation period (control cohort) and 6-month postimplementation period (Pathway cohort). Group comparison: Pearson's chi-square test. Sourced from electronic health record data. ED: emergency department.

As secondary outcome measures, the proportion of participants with >1 medication change was similar in the Pathway and control cohorts (Figure 2). Among the 57 medication changes, 33 events (57%) were a dosage change, 10 (18%) were discontinuations, 9 (16%) were add-on treatments, and 5 (9%) were antidepressant switches. Pathway participants (n=89) were significantly less likely to be referred to behavioral health by their PCPs compared with the control cohort (*P*=.01). In the control cohort (n=90), 4 hospitalizations and 3 ED visits were related to the participant’s behavioral health, while in the Pathway cohort, none were related to the participant’s behavioral health.

#### MDD Severity Improvement

**Figure 3:**
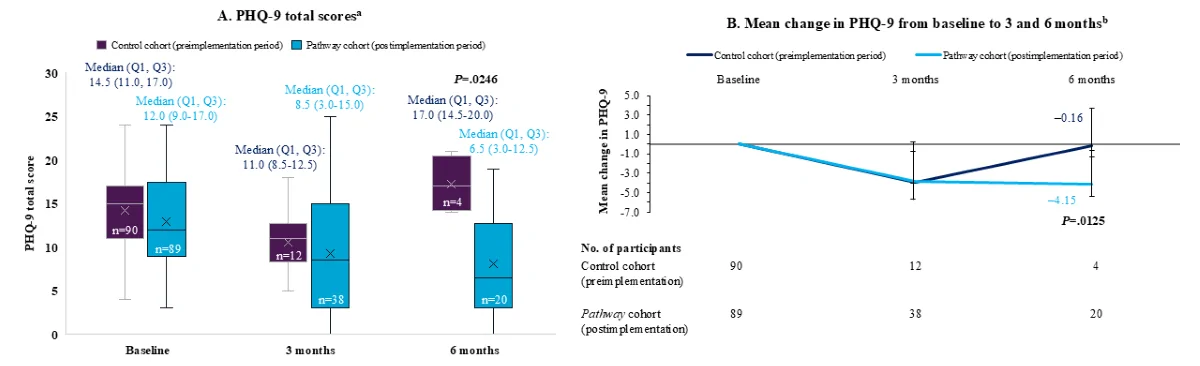
Group comparison: (A) Median Patient Health Questionnaire-9 total scores were compared with the Wilcoxon 2-sample test and (B) mean changes in Patient Health Questionnaire-9 scores from baseline to later time points were compared using a mixed effects model for repeated measures. Boxes show Q1 (bottom), Q3 (top); horizontal line inside each box is the median (Q2) value; whiskers represent the smallest and largest values within 1.5 × IQR; “x” indicates the mean value. (a) Sourced from electronic health record and app data. For the Pathway cohort, “baseline” refers to the scores the patients received on the Patient Health Questionnaire-9 scale on the day they downloaded the Pathway app. For the control cohort, “baseline” refers to the Patient Health Questionnaire-9 scores sourced from the electronic health records on the qualifying visit. (b) For the Pathway cohort, data were sourced from the app. PHQ-9: 9-Item Patient Health Questionnaire; Q: quartile.

In an exploratory analysis of MDD severity improvement, the median PHQ-9 total score at 6 months was significantly lower in the Pathway participants than the control cohort (*P*=.02) (Figure 3A). Mean changes in PHQ-9 score from baseline to 3 months were similar in both cohorts (effect size 0.09; 95% CI –1.98 to 2.16), whereas mean changes in PHQ-9 score from baseline to 6 months were significantly greater in the Pathway cohort than in the control cohort (effect size –4.00; 95% CI –7.32 to –0.67; *P*=.01), indicating continued improvement in MDD severity with use of the Pathway Platform (Figure 3B). In the Pathway cohort, the subset of participants with moderately severe to severe MDD (n=28) showed significantly greater improvement from baseline to last available PHQ-9 score compared with those with mild to moderate MDD (n=45; *P*=.005; Figure S2 in Multimedia Appendix 1).

### Remission and Response

A numerically higher percentage of participants were in remission (PHQ score ≤5) in the Pathway cohort than in the control cohort at 3 months and at 6 months (Table 2). There was no statistically significant difference in remission rates, which may be attributed to the small sample sizes at 3 months (Pathway cohort: n=38, control cohort: n=12) and 6 months (Pathway cohort: n=20, control cohort: n=4). Similarly, rates of MDD response (≥50% reduction in most recent PHQ-9 score) were numerically higher in the Pathway cohort than in controls at 6 months, but differences were not statistically significant. Remission and response rates were similar in the subgroups by disease severity in the Pathway and control cohorts (Table S3 in Multimedia Appendix 1).

**Table 2:** Remission and response rates at 3 months and 6 months in the control cohort (preimplementation period) and Pathway cohort (postimplementation period).

Outcome^a^			Control cohort (preimplementation period) n=90		Pathway cohort (postimplementation period) n=89
		EHR^b^ data		Pathway data	
3 months					
	Remission^c^, n/N (%)	1/12 (8.3)		14/38 (36.8)	
	Response^d^, n/N (%)	4/12 (33.3)		12/38 (31.6)	
6 months					
	Remission, n/N (%)	0/4 (0)		9/20 (45.0)	
	Response, n/N (%)	0/4 (0)		7/20 (35.0)	

^a^Group comparison: Wilcoxon test; n is the number of participants who achieved remission or response; and N is the total number of participants with PHQ-9 remission or response data at 3 and 6 months.

^b^EHR: electronic health record.

^c^Remission rate was defined as the proportion of participants with a PHQ-9 score ≤5 at the end of the study period.

^d^Response rate was defined as the proportion of participants with ≥50% reduction in PHQ-9 score.

### Patient Functioning and Patient–Provider Engagement

As reported by Pathway participants through the app, median WSAS scores, a measure of patient functionality, significantly improved from baseline to 6 months (n=54; median change from baseline –4.0, Q1 –9, Q3 1; 95% CI –7.0 to –1.0]; *P*<.001). Pathway participants experienced statistically significant improvement in median PAM-13 scores from baseline to 6 months (n=54; median change from baseline 4, Q1 0, Q3 9.2; 95% CI 0-4.9; *P<*.001), indicating improved patient-provider engagement (Figure S3A in Multimedia Appendix 1). Cognitive function assessed using median PDQ-D-5 score (with ≥2 assessments) improved from baseline to last available score (n=73; median change from baseline –2, Q1 –6, Q3 0; 95% CI –3 to –1; *P*<.001). For cognitive function assessed using the DSST, the first available score for patients in the Pathway cohort (n=22) within the first 30 days was a median DSST score of 28 (SE 16), and the last available score improved to a median of 43 (SE 22; *P*<.001). Similarly, overall well-being assessed through the WHO-5 showed an improved last available score compared with baseline (n=70; median change from baseline 2, Q1 –1, Q3 6; 95% CI 1-3; *P*<.001; Figure S3B in Multimedia Appendix 1).

### Engagement With Pathway App

Overall, 40 (45%) of the 89 participants in the Pathway cohort logged into the app at least once per week (mean 5.5, SD 7 logins per week during study), with 4 (4%) of the 89 participants opening the app at least once per day (mean 60, SD 49 logins per day during study). Some participants logged in consistently, while others were inactive until the study end for the final round of assessments. All participants in the Pathway cohort completed at least 1 assessment for the PHQ-9, WHO-5, PDQ-D-5, DSST, PAM-13, and WSAS. Approximately 80% or more (87/89 and 71/89) of participants in the Pathway cohort reported medication adherence and medication-associated AEs at least once during the 6-month period (Table S4 in Multimedia Appendix 1). Five participants used the goal-setting feature on the Pathway app. The median number of goals set by these participants was 3 (range 2-5), and the participants achieved a median of 2 goals each (range 0-4). Among the 4 participants who achieved a goal, the mean time to goal achievement was 6 weeks for 2 individuals, and 12 weeks for the other 2 individuals. Among the 17 goals, there were 8 physical goals, 5 social goals, 2 motivational goals, and 1 each of emotional and psychological goals.

### Adverse Effects of MDD Medication Reported by Pathway Participants

Participants who reported sleep problems were prompted to complete the 6-Item PROMIS Sleep Disturbance scale. Of the 89 Pathway participants, 21 completed this scale, resulting in a median baseline score of 22, which decreased to a median score of 20 during the study (*P*=.50; mean follow-up 3.8 months). Further, 21 of 89 Pathway participants reported sexual dysfunction and were prompted to complete the ASEX assessment; 7 participants completed the ASEX, revealing a median baseline score of 19, which decreased to a median score of 17 during the study (*P*=.16) (mean follow-up 3.0 months). A summary of the AEs reported through the app is available in Figure S4 in Multimedia Appendix 1.

## Discussion

### Principal Findings

The Pathway Platform increased engagement between providers and patients with antidepressant-managed MDD and was associated with improved clinical outcomes compared with a preimplementation cohort. PHQ-9 completion was significantly higher (*P*=.01) among the Pathway participants than in the control cohort, suggesting that use of the Pathway Platform led to better MBC and improved performance according to HEDIS guidelines [22]. In surveys of clinicians treating mental disorders, >61% of respondents indicated that they never used standardized scales, and the most common reason cited by clinicians was lack of time (34%) [11,24]. The Pathway Platform can bridge this gap within the real-world primary care setting by providing patients with MDD and their clinicians a tool that offers both education and patient information. It also allows regular monitoring of treatment within the EHR clinical workflow to support clinicians and, through the app, to support patients for improved MDD outcomes. Although the proportion of participants completing ≥2 PHQ-9 assessments was significantly higher in the Pathway cohort compared with the control group, the overall completion rate remained low. This observation is consistent with prior literature showing a range of modest engagement and completion rates with other digital mental health interventions that collect this assessment for longitudinal studies in real-world settings [25-27]. Additionally, individuals with more severe symptoms may face barriers such as low motivation and impaired concentration, which can limit consistent use [28].

The use of PRO assessments during the study was associated with improvements in MDD symptoms and overall functioning. Participants in the Pathway cohort had significantly fewer referrals to behavioral health compared with the control cohort, suggesting less health care resource usage. Remission rate at 3 months and remission and response rates at 6 months were numerically higher among the Pathway cohort; however, these findings warrant cautious interpretation because the differences were not significant, perhaps due to the small sample size. Yet within both cohorts, most participants had not achieved remission at 3 or 6 months, but only 42% (38/90) or 55% (40/89) of participants, respectively, adjusted antidepressant treatment within the observation period. This finding suggests a need for strategies to prompt medication changes for patients with residual symptoms. Subgroup analyses by baseline severity found a significant difference in PHQ usage for participants with mild or moderate MDD, and there was a significant improvement in depression severity among Pathway participants with moderately severe and severe MDD. However, given the observational design and lack of randomization, these findings should be interpreted cautiously and may not be due to causal effects of the Pathway Platform. These results are similar to the findings of a recent meta-regression analysis that reported larger reductions in PHQ-9 scores over 8 weeks of digital health app use among study participants who had severe and moderately severe MDD at baseline compared with participants who had moderate and mild MDD [29]. Similarly, a systematic review of app-based interventions for moderate to severe depression showed a significant medium effect size in studies that assessed the efficacy of such interventions when implemented either as a stand-alone or as an adjunct to conventional treatments [30]. These findings with regard to the use of digital health interventions for MDD parallel those with pharmacotherapy for MDD, from which patients with very severe MDD derive greater benefit from antidepressant treatment than patients with less severe symptoms [31]. Notably, higher platform engagement was observed among individuals with mild to moderate MDD, whereas greater symptom improvement was seen in those with more severe baseline symptoms. Patients with moderately severe and severe MDD may have engaged less with the app due to symptom-related barriers such as anhedonia, concentration difficulty, and hopelessness, while at the same time, these patients may have greater potential for measurable symptom reduction. These observations are consistent with prior findings in digital and pharmacological interventions [28,30,31]. Additionally, although antidepressant therapy may have contributed to symptom improvement, baseline medication use was largely comparable between the Pathway and control cohorts, suggesting that differences in pharmacological treatment alone are unlikely to account for the observed outcomes.

Patient engagement with providers was improved among Pathway participants, indicating that the platform is a viable tool for recording data and maintaining this communication in primary care. Another web-based application (REDCap) that integrates with EHRs was evaluated for usability to collect digitized self-assessments in a Canadian primary care setting to inform clinical decisions [32]. Results showed that electronic data collection resulted in significant time savings, real-time information relay, and improved data quality [32]. Additionally, there was a 4-fold increase in clinicians adopting this pathway, as it supports integration of MBC and SDM within a primary care clinical workflow [32]. A meta-analysis showed that mental health apps with engagement features, such as prompts, reminders, check-ins, and educational features, tend to improve adherence and clinical outcomes for patients [33]. The current version of the Pathway Platform was developed based on input from people with MDD and their care teams [10], incorporating the need for real-time data flow between app and EHR and educational programming intended for both patients and care teams. The Pathway app also prompts patients to complete validated assessment scales every 2 weeks, which is consistent with the present APA recommendations for monitoring treatment response during an acute phase [4,6]. A recent survey of a large primary care system in the United States found that almost 3 of 4 providers were extremely likely to use the PHQ-9 for screening for MDD or postpartum depression, indicating that this scale is broadly accepted in clinical practice [34]. The safety feature of the Pathway Platform enables continuous reporting of medication-associated AEs, giving clinicians valuable insights into patients’ experiences with issues like sleep disturbances, sexual dysfunction, and other concerns between visits. By facilitating ongoing symptom and AE tracking, the platform promotes active patient participation in care and enables more responsive treatment adjustments. HCPs consider AE tracking to be clinically valuable, partly because continuous and direct input from the patient provides a clinically useful view of how each patient’s condition changes over time, thereby supporting better clinical decision-making [10]. Thus, the platform offers educational support and patient information integrated into the EHR workflow, which helps meet the needs of clinicians and patients in real-world health care settings.

The Pathway Platform and its associated app provide a potential tool to address challenges faced in implementing MBC and SDM in the primary care setting, where clinicians generally lack time to administer periodic validated assessments for people with MDD. Goal setting is a practical approach to applying the principles of SDM in clinical practice. The goal setting and tracking feature of the Pathway Platform was designed to help patients focus on their goals and find a constructive purpose*.* This feature may support clinical conversations, help patients focus on their treatment goals, and enable primary care providers to better assess whether patients are making adequate progress toward those goals, thereby enhancing patient-provider engagement and ultimately patient outcomes [6,10]. Additionally, platforms such as Pathway, with a user-centered design, can potentially improve adherence, resulting in improved patient-provider engagement and informed decisions in patient care. Future research is needed to explore the long-term clinical impact, cost-effectiveness, and comparative effectiveness of digital tools for MBC adoption to further enhance the clinical significance of the Pathway Platform*.*

### Limitations

In this study, patients were not distributed by randomization to the preimplementation and postimplementation periods because this was a cohort study in which historical data were used for the control cohort, resulting in demographically diverse study arms. An imbalance in baseline characteristics was evident between the control and Pathway cohorts in the proportions of female and younger participants. Although the cohorts were selected by the same eligibility criteria, the size of the control cohort was limited by the number of patients with available PHQ measures and a medication adjustment. Further, the Pathway cohort included a higher proportion of female participants, a trend often observed in consented research, and tended to be slightly younger, likely reflecting greater familiarity with digital tools. However, it is worth noting that the patient populations had similar median baseline PHQ-9 scores. Although these results may not be generalizable to the general population, more patients in the intervention arm were non-Hispanic Black, indicating that the results can provide information on the use of digital health technology by people according to self-reported racial or ethnic categories, which is not a common finding of many health technology studies [35]. This study was not designed to evaluate treatment effectiveness, and the observational design limits the ability to attribute changes in PHQ-9 scores directly to the Pathway Platform. Accordingly, the MDD severity improvement reported should be interpreted as descriptive observations in a real-world setting supporting the potential clinical utility of the platform rather than causal effects. Another limitation of this study is the small sample size and high dropout rate, which may limit generalizability and introduce attrition bias, affecting the reliability of the results. In addition, remission and response outcomes were available only for a subset of patients in both cohorts due to the limited availability of PHQ-9 scores, which may further limit the generalizability of these findings.

### Conclusions

The implementation of the Pathway Platform app in our PCP network, the largest HCP organization in Illinois, increased use of validated assessments and improved patient-provider engagement during an MDD episode. Broader use of MBC was facilitated by the improved integration of the HEDIS-recognized PHQ-9 assessment into the clinical workflow by the Pathway Platform. Significant improvements in symptomatic, cognitive, and functional scales were recorded among participants in the Pathway cohort during this 6-month study. The Pathway app facilitated SDM, incorporating data provided by the patient to the HCP, for better management of MDD. Overall, these findings support the benefits of using the Pathway Platform for MDD management, MBC, and SDM in patients with MDD. Further research is warranted to evaluate the long-term impact and cost-effectiveness of the Pathway Platform as well as its comparative effectiveness among other digital tools in primary care.

## Appendix

Multimedia Appendix 1 Additional tables and figures.

## Abbreviations

AAH: Advocate Aurora Health
AE: adverse event
APA: American Psychiatric Association
ASEX: Arizona Sexual Experience Scale
DSST: Digit Symbol Substitution Test
ED: emergency department
EHR: electronic health record
EMR: electronic medical record
HCP: health care provider
HEDIS: Healthcare Effectiveness Data and Information Set
iCHECK-DH: Guidelines and Checklist for the Reporting on Digital Health Implementations
MBC: measurement-based care
MDD: major depressive disorder
PAM-13: 13-Item Patient Activation Measure
PCP: primary care provider
PDQ-D-5: 5-Item Perceived Deficits Questionnaire–Depression
PHQ-2: 2-Item Patient Health Questionnaire
PHQ-9: 9-Item Patient Health Questionnaire
PRO: patient-reported outcome
PROMIS: Patient-Reported Outcomes Measurement Information System
SDM: shared decision-making
SMART: Substitutable Medical Applications, Reusable Technologies
SOC2-compliant: compliant with Systems and Organization Controls 2 criteria
STROBE: Strengthening the Reporting of Observational Studies in Epidemiology
WHO-5: 5-Item World Health Organization Well-Being Index
WSAS: Work and Social Adjustment Scale

## Data Availability

The datasets generated or analyzed during this study are available from the corresponding author on reasonable request.
