# Supplementary material for: Real-World Implementation and Evaluation of a Digitally Enabled Care Pathway to Improve Depression Management in Primary Care: Controlled Pre-Post Analysis of the Pathway Platform
Source: JMIR Form Res. 2026 Aug 6;10:e93024. doi: 10.2196/93024 (PMC13494522; doi:10.2196/93024)
Supplement: Supplementary file 1 [file formative_v10i1e93024_app1.docx]

**Supplementary material for:**

**Real-World Implementation and Evaluation of a Digitally Enabled Care Pathway to Improve Depression Management in Primary Care: A Controlled Pre–Post Analysis of the *Pathway Platform***

Khatib R, et al

**Table S1. Study assessments**

| **Assessment** | **Parameter measured** | **Details of assessment** | **Range of scores** | **Implication of scores** |
| --- | --- | --- | --- | --- |
| **PHQ-9** | Depressive symptoms (DSM-IV) | Questions based on how much each symptom bothered them in the last 2 weeks | Each question is rated from 0 (not at all) to 3 (nearly every day), with a total score ranging from 0 to 27 | Higher score indicates greater severity |
| **PDQ-D-5** | Cognitive symptoms | 4 subscales with 20 items rated using a 5-point ordinal response to indicate frequency of specific cognitive problems in the past 7 days | Scores range from 0 (never in the past 7 days) to 4 (very often—more than once a day), with a total of 0-80 | Higher score indicates greater perceived cognitive dysfunction |
| **WHO-5** | Overall well-being | 5 mood-related questions | Rated 5 (all the time) to 0 (none of the time) | Higher total score indicates better overall well-being |
| **DSST** | Cognitive performance | Participants to match symbols to numbers as per a prespecified key | 0 to 133 | Higher score indicates better cognitive performance |
| **PAM-13** | Patient activation | 13 questions that measure participants’ engagement with their health and MDD | Scores ranging from 1 (strongly disagree) to 4 (strongly agree), and 0 (not applicable); total 0-100 | Higher scores indicate better activation |
| **WSAS** | Functioning | Assesses impaired work and social functioning through 5 questions | Rated 0 (no impairment) to 8 (severe impairment) | Higher total score indicates impaired functioning |

DSM-IV: *Diagnostic and Statistical Manual of Mental Disorders, Fourth Edition*; DSST: Digit Symbol Substitution Test; MDD: major depressive
disorder; PAM-13: 13-Item Patient Activation Measure; PDQ-D-5: 5-Item Perceived Deficits Questionnaire–Depression; PHQ-9: 9-Item Patient
Health Questionnaire; WHO-5: 5-Item World Health Organization Well-Being Index; WSAS: Work and Social Adjustment Scale.

**Table S2. Utilization of PHQ in subgroups defined by baseline MDD severity**

|  | **N (%) patients with  ≥2 PHQ assessments^a^** | ***P* value** |
| --- | --- | --- |
| **Mild to moderate MDD** |  |  |
| Preimplementation control cohort (n=54) | 19 (35.0) | NA |
| *Pathway* participants (n=47) | 27 (57.0) | .03 |
| **Moderately severe to severe MDD** |  |  |
| Preimplementation control cohort (n=36) | 16 (44.0) | NA |
| *Pathway* participants (n=42) | 22 (52.0) | .49 |

^a^Includes both PHQ-2 and PHQ-9 assessments, sourced from the EHR data.

Group comparison: Pearson chi-square test.

EHR: electronic health records; MDD: major depressive disorder; NA: not applicable; n: number of participants; PHQ-2: 2-Item Patient Health Questionnaire; PHQ-9: 9-Item Patient Health Questionnaire.

**Table S3. Remission and response at 3 months and 6 months in *Pathway* and control cohorts defined by baseline MDD severity**

|  | **Mild or moderate MDD** | | | **Moderately severe or severe MDD** | | |
| --- | --- | --- | --- | --- | --- | --- |
|  | **Control cohort n=54** | ***Pathway* cohort  n=47** | ***P* value** | **Control cohort n=36** | ***Pathway* cohort  n=42** | ***P* value** |
|  | **EHR data** | ***Pathway* data** |  | **EHR data** | ***Pathway* data** |  |
| **3 months** |  |  |  |  |  |  |
| Remission,^a^ n/N (%) | 0/6 (0) | 10/24 (41.7) | .07 | 1/6 (16.7) | 4/14 (28.6) | 1.00 |
| Response,^b^ n/N (%) | 1/6 (16.7) | 8/24 (33.3) | .64 | 3/6 (50.0) | 4/14 (28.6) | .61 |
| **6 months** |  |  |  |  |  |  |
| Remission,^a^ n/N (%) | 0/1 (0) | 7/13 (53.9) | 1.00 | 0/3 (0.0) | 2/7 (28.6) | 1.00 |
| Response,^b^ n/N (%) | 0/1 (0) | 4/13 (30.8) | 1.00 | 0/3 (0.0) | 3/7 (42.9) | .48 |

Group comparison: Wilcoxon test.

^a^Remission rate was defined as the proportion of participants with a PHQ-9 score ≤5 at the end of the study period.

^b^Response rate was defined as the proportion of participants with ≥50% reduction in PHQ-9 score.

EHR: electronic health records; MDD: major depressive disorder; N: total number of participants with PHQ-9 remission/response data at 3 and 6 months; n: number of participants who achieved remission/response; PHQ-9: 9-Item Patient Health Questionnaire.

|  | **Pathway**  **assessments** | **Prompted to complete** | **Percentage of participants who completed at least 1 assessment in 6 months, n (%)** | **Median (IQR) number of assessments completed per participant during  6 months** |
| --- | --- | --- | --- | --- |
| Depression, well-being, and cognition | PHQ-9 | Every 2 weeks; 3-day window | 89 (100.0) | 4 (4) |
|  | WHO-5 | Every 2 weeks; 3-day window | 89 (100.0) | 3 (4) |
|  | PDQ-D-5 | Every 2 weeks; 3-day window | 89 (100.0) | 4 (4) |
|  | DSST | Daily | 89 (100.0) | 7 (12) |
| Patient-provider engagement | PAM-13 | Baseline, 6 months; 30-day window | 89 (100.0) | 2 (1) |
|  | WSAS | Baseline, 6 months; 30-day window | 89 (100.0) | 2 (1) |
|  | CollaboRATE | Baseline, 6 months; 30-day window | 78 (88.0) | 2 (1) |
| Medication adherence and medication-associated AEs | Medication  adherence | Daily | 87 (98.0) | 61 (102) |
|  | AEs | Daily | 71 (80.0) | 9 (21) |
|  | ASEX | *When reporting side effects related to sexual dysfunction (n=21)* | 9 (10.0) | 5 (8) |
|  | PSD-6 | *When reporting side effects related to sleep (n=45)* | 22 (25.0) | 7 (6) |
| Goals | Goals set | Goal can be set at any time during the study period | 5 (6.0) | 3 (3) |
|  | Goal progress | Prompted if goal is set, depending on the start date of the goal | 4 (4.0) | 19 (20) |

**Table S4. Engagement with *Pathway* app and completion of assessments**

Sourced from app data.

AEs: adverse events; ASEX: Arizona Sexual Experience Scale; DSST: Digit Symbol Substitution Test; PAM-13: 13-Item Patient Activation Measure; PDQ-D-5: 5-Item Perceived Deficits Questionnaire–Depression; PHQ-9: 9-Item Patient Health Questionnaire; PSD-6: Patient-Reported Outcomes Measurement Information System (PROMIS) Sleep Disturbance 6a; WHO-5: 5-Item World Health Organization Well-Being Index; WSAS: Work and Social Adjustment Scale.

**Figure S1. Participant disposition in *Pathway* cohort (postimplementation)**

MDD: major depressive disorder; n: number of participants; PCP: primary care provider; PHQ-9: 9-item Patient Health Questionnaire.

**Figure S2. Median PHQ-9 scores from baseline to last available score stratified by MDD severity among the *Pathway* cohort**

Boxes show Q1 (bottom), Q3 (top); horizontal line inside each box is the median (Q2) value; whiskers represent the smallest and largest values within 1.5 × IQR; “x” indicates the mean value.

Score comparison: Wilcoxon test.

Sourced from app data.

IQR: interquartile range; MDD: major depressive disorder; n: number of participants; PHQ-9: 9-Item Patient Health Questionnaire; Q: quartile.

**Figure S3. Median functional improvement and patient-provider engagement scores postimplementation among the *Pathway* cohort**

| **A. Change from baseline at 6 months in WSAS and PAM-13** | **B. Change from baseline at last available score in WHO-5 and PDQ-D-5** |
| --- | --- |
|  | |

Boxes show Q1 (bottom), Q3 (top); horizontal line inside each box is the median (Q2) value; whiskers represent the smallest and largest values within 1.5 × IQR; “x” indicates the mean value; colored dots represent outliers (values beyond 1.5 × IQR).

Score comparison: Wilcoxon test.

Sourced from app data.

IQR: interquartile range; n: number of participants; PAM-13: 13-Item Patient Activation Measure; PDQ-D-5: 5-Item Perceived Deficits Questionnaire–Depression; Q: quartile; WHO-5: World Health Organization Well-Being Index; WSAS: Work and Social Adjustment Scale**.**

**Figure S4. Medication-associated adverse events reported through the *Pathway* app by participants**
